# A Novel Scale for Diagnosis of Pulmonary Ground‐Glass Nodules: A Multicenter and Ambispective Cohort Study

**DOI:** 10.1111/crj.70027

**Published:** 2024-11-09

**Authors:** Minhao Yu, Yalin Cheng, Tao Wen, Liming Zhang, Xiubo Wei, Yonghong Wang, Jiang Du, GuangKe Xie, Lei Bi

**Affiliations:** ^1^ Department of Thoracic Surgery Chengdu BOE Hospital Chengdu China; ^2^ Department of Thoracic Surgery Mianyang 404 Hospital Mianyang China; ^3^ Department of Clinical Laboratory Third Hospital of Mianyang, Sichuan Mental Health Center Mianyang China; ^4^ Department of Thoracic Surgery Sichuan Science City Hospital Mianyang China; ^5^ Department of Thoracic Surgery Chengdu Pidu District Hospital of Traditional Chinese Medicine Chengdu China; ^6^ Department of Cardiothoracic Surgery, Bishan Hospital of Chongqing Bishan Hospital of Chongqing Medical University Chongqing China

**Keywords:** diagnostic efficiency, GGNs, lung cancer screening, prediction model, screening scale

## Abstract

**Background:**

A screening tool was devised to aid the diagnosis and treatment of ground‐glass nodules (GGNs).

**Methods:**

The current ambispective cohort study included retrospective collation of 20 variables synthesizing a patient's clinical characteristics, serum tumor markers, and CT results, which allowed division into noninvasive (benign, atypical adenomatous hyperplasia, and adenocarcinoma in situ) and invasive (minimally invasive and invasive adenocarcinomas) tumors to build a prediction nomogram and GGN screening scale. The model was verified internally. A prospective cohort of patients was randomly divided by envelope method into those assessed by the GGN screening scale and those assessed via CT values. The diagnostic efficiencies were compared to allow external verification of the model.

**Result:**

A total of 223 patients with 225 GGNs were recruited into the retrospective cohort between January 2021 and December 2022. Multivariable analysis showed sex, diameter, air bronchogram, and vessel convergence sign to be independent factors for prediction of noninvasive and invasive GGNs. Internal verification showed the model had a sensitivity of 70.7% and specificity of 75.0% with the Youden index at 0.457 and area under the curve (AUC) of 0.793 (95% CI: 0.734–0.852). Calibration curves indicated good internal stability (*p* = 0.357). Between January 2023 and March 2023, 147 patients with 148 GGNs were recruited into the prospective cohort. External verification showed the model had a sensitivity of 92.4% and specificity of 40.0% with the Youden index at 0.324 and AUC of 0.678 (95% CI: 0.509–0.847). Calibration curves indicated good external stability (*p* = 0.088). The scale was shown to have a sensitivity of 75.00%, specificity of 37.50%, positive predictive value of 91.53%, negative predictive value of 14.29%, and accuracy of 71.25%.

**Conclusion:**

The GGN screening scale has high sensitivity and accuracy, making it suitable for diagnosis of GGNs.

## Graphical abstract

A new way to diagnose ground‐glass nodules is presented.

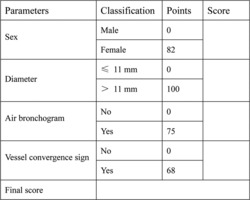

## 1 Introduction

Ground‐glass nodules (GGNs) represent a radiological finding from computed tomography (CT) scans and consist of a hazy opacity that does not obscure the underlying bronchial structures or pulmonary vessels [1]. Pure ground‐glass nodules (pGGNs) are found in combination with mixed ground‐glass nodules (mGGNs), which have either a ground glass component and a consolidated component (lung windows), forming heterogeneous GGNs, or a ground glass component and a solid component (mediastinal windows), forming part‐solid nodules (PSN) [2].

GGNs represent radiological findings of different pathological processes, and some correlation is shown between CT images and pathological types. pGGNs have been reported to be often benign with only about 18% being malignant [3]. Atypical adenomatous hyperplasia (AAH) tends to produce a pGGN of less than 5 mm [1, 4, 5] and adenocarcinoma in situ (AIS) a pGGN from 6 to 30 mm [5]. Minimally invasive adenocarcinoma (MIA) often produces a mGGN with a solid component larger than 5 mm and is accompanied by an abnormality of the pulmonary vein, air bronchogram, and pleural indentation [6]. pGGNs of greater than 15 mm in diameter with nodularity or high pixel attenuation (> −472 HU) are more likely to be invasive adenocarcinoma (IA) [7]. A mGGN with a solid part larger than 5 mm or a pGGN larger than 30 mm is usually a MIA, lepidic predominant adenocarcinoma, or IA [5, 8]. IA has been reported among part‐solid nodules [9]. However, the solid area may represent a benign or fibrous scar harboring a stromal invasive component [10]. Therefore, GGNs remain a diagnostic challenge on account of their malignant potential and heterogeneous characteristics, and a systematic approach is necessary to ensure correct diagnosis and optimal management.

Low‐dose spiral CT (LDCT) is an accepted screening method for reducing lung cancer mortality. The National Lung Screening Trial (NLST) in the United States reported a 20.0% reduction in lung cancer mortality and a 6.7% reduction in all‐cause mortality after LDCT screening. Consequently, LDCT screening for lung cancer is widely used in the United States and China [11, 12], and increasing numbers of GGNs are found. National Comprehensive Cancer Network guidelines (Version 3.2022) for nonsmall cell lung cancer (NSCLC) recommend dynamic observation by scanning LDCT every 6 to 12 months for pGGNs and every 3 to 6 months for mGGNs larger than 6 mm [13]. This recommendation has achieved worldwide acceptance to reduce the risk of invasive procedures and to prolong survival.

However, controversies remain regarding LDCT for GGN screening, such as excessive screening, prolonged follow‐up time, high false‐positive rate, and radiological hazard. The NLST gave false positive screening rates of 96.4%, similar to those of chest X‐ray (94.5%). As a result, 2.58% of patients with positive LDCT results underwent invasive procedures without having confirmed lung cancer [14]. LDCT has a low specificity rate on pulmonary nodules less than 3 cm because of the lack of lobulation, spicule, vacuole, bronchogram, and vessel convergence sign and pleural indentation [15]. It is also the case that lung cancer screening did not change participants' lung cancer or smoking‐related disease risk perceptions: Negative screening results did not appear to decrease risk perceptions nor provide false reassurance to smokers [16]. Furthermore, screening exacerbates patient anxiety when pulmonary nodules are detected and national differences in medical insurance produce nonuniform LDCT cost‐effectiveness, meaning that increased screening frequency levies an economic burden [17]. In addition, the timing of surgical intervention is also controversial. Resection of invasive lesions, MIA and IA, is widely accepted to produce excellent surgical results, but the influence of surgery on outcomes of preinvasive lesions, AAH and AIS, remains unclear. Sugi et al. [18] and Koike et al. [19] have suggested that sublobe resection may be successful for preinvasive lesions, whereas van Schil et al. [20] recommended CT follow‐up rather than immediate resection, due to the indolent nature of the lesions. Such findings emphasize the importance of early differentiation between noninvasive (benign and preinvasive) and invasive GGNs during preoperative examinations and the avoidance of overdiagnosis.

The clinical experience of the current authors inclines us towards surveillance and observation for noninvasive and surgical intervention for invasive GGNs. The aim of the present work was to create a GGN screening scale for diagnosis of CT‐detected GGN pathological type to guide treatment. To the best of our knowledge, there is no such preexisting scale.

## 2 Materials and Methods

### 2.1 Trial Design

**FIGURE 1:**
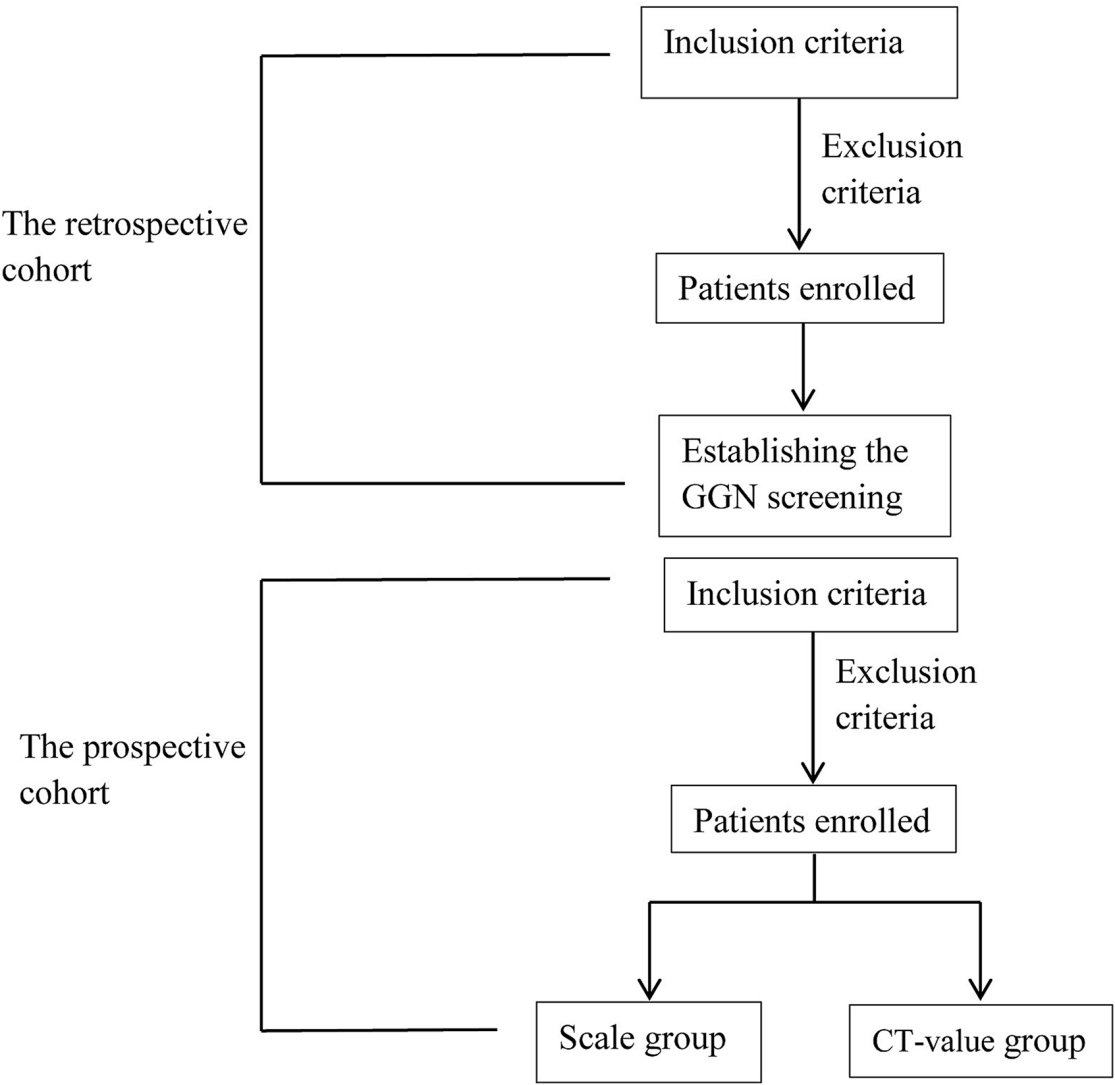
The flow chart of hypothetical system.

The current ambispective cohort study encompassed a retrospective cohort using data from which a prediction nomogram was developed, a scale was constructed, and the model was verified internally. A second prospective cohort was enrolled to test the diagnostic efficiency of the scale for external verification of the model (see Figure 1 for flow chart). The study was conducted in accordance with the Declaration of Helsinki, as revised in 2000. The study was approved by the Ethics Committee of the Chengdu BOE Hospital (No. 2022004). Informed consent was obtained from all participants and their legal guardians (clinical trial registry number: ChiCTR2300070812).

### 2.2 Establishment of the GGN Screening Scale

#### 2.2.1 Patients (Retrospective Cohort)

Patients were recruited from Chengdu BOE Hospital, Bishan Hospital of Chongqing Medical University, Sichuan Science City Hospital and Chengdu Pidu District Hospital of Traditional Chinese Medicine. The required sample size was at least 10 events per candidate predictor parameter [21]. Inclusion criteria were as follows: > 18 years old; high resolution CT detection of GGNs; and diagnosis confirmed by pathological biopsy resulting from surgery, bronchoscopy, or percutaneous cytological examination (complete tumor sampling is mandatory for the diagnosis of AAH, AIS, or MIA [8]). Exclusion criteria were as follows: incomplete clinical data and patients who were not followed up, treated, or biopsied due to advanced age, cardiopulmonary dysfunction, severe complications, or other reasons.

#### 2.2.2 Data Collection

Three types of data were collated: clinical characteristics, including gender, age, history of smoking, history of pulmonary disease, family history of lung cancer, and environmental and occupational exposure; serum tumor markers (TMs), including carcinoembryonic antigen (CEA), neuron‐specific enolase (NSE), progastrin‐releasing peptide (proGRP), CyFRA21‐1, and squamous cell carcinoma antigen (SCCA); and CT features, including diameter, mean CT value in Hounsfield units (HU), solid component size, consolidation/tumor ratio (CTR), lobulation sign, spicule sign, air bronchogram, vessel convergence sign, and pleural indentation.

#### 2.2.3 Prediction Model and GGN Screening Scale

Univariable and multivariable analyses of the data were performed, and results showing statistical significance for the prediction of noninvasive and invasive GGNs were used to construct a nomogram. Four items were incorporated into the GGN screening scale: parameters, classification, points, and score. Points and classification of each parameter in the nomogram correspond to the same items in the scale. The score of each parameter on the scale was derived from the point of corresponding classification in the nomogram. All scores for each parameter were added to derive the final score. Final score cut‐off point was determined by the first quartile.

### 2.3 The Diagnostic Efficiency of the Scale

#### 2.3.1 Patients (Prospective Cohort)

Patients were recruited from Chengdu BOE Hospital, Bishan Hospital of Chongqing Medical University, Sichuan Science City Hospital and Chengdu Pidu District Hospital of Traditional Chinese Medicine. Patients with CT‐detected GGNs were randomly divided by envelope method into a group that was assessed using the scale and a group that was assessed on the basis of CT‐value. Inclusion criteria were as follows: > 18 years old; high resolution CT detection of GGNs; and diagnosis confirmed by pathological biopsy resulting from surgery, bronchoscopy, or percutaneous cytological examination (complete tumor sampling is mandatory for the diagnosis of AAH, AIS or MIA [8]). Exclusion criteria were as follows: incomplete clinical data and patients who were not followed up, treated, or biopsied due to advanced age, cardiopulmonary dysfunction, severe complications, or other reasons.

#### 2.3.2 Invasive Nodule Criteria

Invasive nodules were defined from the final score > the cut‐off point according to the GGN scale for the scale group or as mean CT value > −368.25 HU for the CT group. Yoshida et al. [22] have previously considered GGNs to be invasive nodules when the mean CT value > −368.25 HU. Test nodules were independently classified and diagnosed by an experienced radiologist and a senior thoracic surgeon who were blinded to patients' information. GGNs were confirmed by pathological biopsy and mandatory tumor sampling for the diagnosis of AAH, AIS, or MIA [8].

#### 2.3.3 Sample Size

The sample size was calculated based on sensitivity using the following formula: $n={\left(\frac{Z_{1-\frac{\alpha }{2}}\times \sqrt{p\times \left(1-p\right)}}{\delta }\right)}^{2}$. The statistical significance level (α) of hypothesis testing was 0.05, and the *Z*_1‐α/2_ = 1.96. The value of *p* represents the sensitivity of both groups, and δ represents the confidence interval width and was set to 0.1.

### 2.4 Statistical Analysis

SPSS 22.0 (IBM Corp., Armonk, NY, United States) software was used for statistical analysis. Continuous data are expressed as mean ± standard deviation, and *t*‐test was used to compare normally distributed data. The Mann–Whitney *U* test was used to compare nonnormally distributed data. Categorical data are expressed as counts and percentages and were compared by chi‐square or Fisher exact tests. All continuous variables were divided into two levels for binary logistic regression analysis by cut‐off points of their receiver operating characteristic (ROC) curves. Indicators with statistically significant results from univariable analysis were incorporated into logistic regression for multivariable analysis to screen for risk factors predicting invasive GGNs. The nomogram was constructed using R programming language, Version 4.2.2. Internal validation was performed by bootstrapping (1:1000). Excel software was used to draw calibration curves. A value of *p* < 0.05 was considered to indicate statistical significance.

## 3 Results

### 3.1 Establishment of the GGN Screening Scale

#### 3.1.1 Patient Characteristics

A total of 223 patients, 86 men and 137 women, with 225 GGNs were recruited between January 2021 and December 2022, a number that exceeded the required minimum sample size. Mean age of the noninvasive group was 55.35 ± 12.42 years and of the invasive group 56.85 ± 12.39 years (*p* = 0.868, age range of whole cohort: 21–82 years). The cut‐off point was 63.5 years (Table 1).

**TABLE 1:** The cut‐off point of age by ROC curve.

Age (years old)	AUC	Youden index
36.5	0.534	0.114

Abbreviations: AUC, area under the curve; ROC, receiver operating characteristic.

No significant differences were found between the two groups in history of smoking, history of lung disease, family history of lung cancer, occupational or environmental factors, or location of tumor. Significant differences were found in sex, age, histopathology, pathological type, and stage (Table 2).

**TABLE 2:** Clinical characteristics of patients in the retrospective cohort.

Items	Noninvasive (*n* = 68)	Invasive (*n* = 157)	*p*
Sex			0.033
Male	31	57	
Female	35	100	
Age (years old)			0.017
< 64	51	101	
≥ 64	15	53	
History of smoking			0.079
Yes	16	32	
No	50	125	
History of lung disease			0.326
COPD	0	8	
TB	0	0	
Pulmonary fibrosis	0	5	
Family history of lung cancer	0	3	0.555
Occupational or environmental factors	0	1	> 0.999
Histopathology			< 0.001
Benign	27	2	
Adenocarcinoma	41	155	
Squamous	0	0	
Pathological type			< 0.001
Benign	28	1	
AAH	1	0	
AIS	39	0	
MIA	0	59	
IA	0	97	
Location of tumor			0.980
RUL	25	63	
RML	6	13	
RLL	9	17	
LUL	19	42	
LLL	9	22	
Stage			< 0.001
AAH	1	0	
AIS	39	1	
IA1	0	58	
IA2	0	50	
IA3	0	29	
IB1	0	14	
IB2	0	1	
IIB	0	2	
IIIB	0	1	

Abbreviations: AAH, atypical adenomatous hyperplasia; AIS, adenocarcinoma in situ; COPD, chronic obstructive pulmonary disease; IA, invasive adenocarcinoma; LLL, left lower lung; LUL, left upper lung; MIA, minimally invasive adenocarcinoma; RLL, right lower lung; RML, right middle lung; RUL, right upper lung; TB, tuberculosis.

#### 3.1.2 TMs Between the Two Groups

Variability of instruments used at different centers precluded a comparison of TM counts. Instead, TM results were divided into three levels: normal, less than twofold above normal value and more than twofold above the normal value. No significant differences were found between the groups (Table 3).

**TABLE 3:** Comparison of TMs between the two groups.

TMs	Noninvasive (*n* = 68)	Invasive (*n* = 157)	*p*
CEA			0.411
Normal	66	148	
Rise < 2‐fold	2	5	
Rise ≥ 2‐fold	0	4	
NSE			0.639
Normal	64	150	
Rise < 2‐fold	4	6	
Rise ≥ 2‐fold	0	1	
proGRP			0.545
Normal	62	138	
Rise < 2‐fold	5	18	
Rise ≥ 2‐fold	1	1	
SCCA			0.299
Normal	65	151	
Rise < 2‐fold	2	6	
Rise ≥ 2‐fold	1	0	
CyFRA21–1			0.132
Normal	67	153	
Rise < 2‐fold	0	4	
Rise ≥ 2‐fold	1	0	

Abbreviations: CEA, carcinoembryonic antigen; NSE, neuron‐specific enolase; proGRP, progastrin‐releasing peptide; SCC, squamous cell carcinoma antigen; TMs, tumor markers.

#### 3.1.3 CT Features

Mean GGN diameter for the noninvasive group was 10.46 ± 5.92 mm, and for the invasive group, 13.62 ± 6.36 mm (*p* = 0.021). Mean CT value for the noninvasive group was −460.84 ± 214.52 HU, and for the invasive group, −396.99 ± 199.99 HU (*p* = 0.869). Mean solid component size for the noninvasive group was 4.70 ± 5.91 mm, and for the invasive group, 5.64 ± 4.23 mm (*p* = 0.883). Mean CTR for the noninvasive group was 39.98% ± 21.16%, and for the invasive group, 41.36% ± 27.91% (*p* = 0.016).

Cut‐off points were diameter: 11.15 mm; mean CT value: −430.56 HU; solid component size: 3.87 mm, and CTR: 33.71% (Table 4). Intergroup comparisons of CT characteristics are in Table 5.

**TABLE 4:** The cut‐off point of diameter, mean CT value, solid component size, and CTR by ROC curve.

	Cut‐off point	AUC	Youden index
Diameter (mm)	11.15	0.673	0.325
Mean CT value (HU)	−430.56	0.613	0.226
Solid component size (mm)	3.87	0.607	0.244
CTR (%)	33.71	0.516	0.107

Abbreviations: AUC, area under the curve; CTR, consolidation/tumor ratio; ROC, receiver operating characteristic.

**TABLE 5:** Comparison of CT features between the two groups.

CT features	Noninvasive (*n* = 68)	Invasive (*n* = 157)	*p*
Diameter			<0.001
≤ 11.15 mm	52	69	
> 11.15 mm	16	88	
Mean CT value			0.003
≤ −431	47	74	
> −431	21	83	
Solid component size			0.001
≤ 3.9 mm	43	62	
> 3.9 mm	25	95	
CTR			0.009
≤ 34%	41	64	
> 34%	27	93	
Lobulation sign	16	69	0.006
Spicule sign	12	70	<0.001
Air bronchogram	18	85	<0.001
Vessel convergence sign	33	122	<0.001
Pleural indentation	12	57	0.007

Abbreviations: CTR, consolidation/tumor ratio; mm, millimeter.

#### 3.1.4 Multivariate Regression Analysis and Risk Prediction Model

Multivariate analysis showed that sex, diameter, air bronchogram, and vessel convergence sign were independent risk factors (Table 6). The model was calculated as follows: $P=\frac{e^{x}}{1+e^{x}}$, where the *e* is the natural logarithm, *x* = 1.294–0.982 × sex (male = 1, female = 0) − 1.024 × diameter (≤11.15 mm = 1, >11.15 mm = 0) + 0.927 × air bronchogram (yes = 1, no = 0) + 0.875 × vessel convergence sign (yes = 1, no = 0).

**TABLE 6:** Multivariate analysis on risk variables.

	β	SE	Wald	HR (95% CI)	*p*
Sex (male vs. female)	−0.982	0.379	6.726	0.375 (0.178–0.787)	0.010
Age (≥ 64 years old vs. < 64)	0.004	0.402	0.000	1.004 (0.457–2.208)	0.991
Diameter (≤ 11.15 mm vs. > 11.15 mm)	−1.024	0.455	5.072	0.359 (0.147–0.876)	0.024
Mean CT value (≤ −431 vs. > −431)	−0.567	0.365	2.408	0.567 (0.277–1.161)	0.121
Solid component size (> 3.9 mm vs. ≤ 3.9 mm)	0.448	0.593	0.571	1.565 (0.489–5.005)	0.450
CTR (≤ 34% vs. > 34%)	−0.654	0.543	1.453	0.520 (0.179–1.506)	0.228
Lobulation sign (yes vs. no)	0.331	0.407	0.663	1.393 (0.627–3.091)	0.416
Spicule sign (yes vs. no)	0.623	0.478	1.699	1.865 (0.731–4.762)	0.192
Air bronchogram (yes vs. no)	0.927	0.401	5.344	2.526 (1.151–5.542)	0.021
Vessel convergence sign (yes vs. no)	0.875	0.372	5.540	2.399 (1.158–4.973)	0.019
Pleural indentation (no vs. yes)	−0.654	0.501	1.701	0.520 (0.195–1.389)	0.192
Constant	1.294	0.572	5.119	3.648	0.024

Abbreviations: CI, confidence interval; CTR, consolidation/tumor ratio; HR, harzard ratio; SE, standard error; mm, millimeter.

#### 3.1.5 Diagnostic Value and Model Validation

**FIGURE 2:**
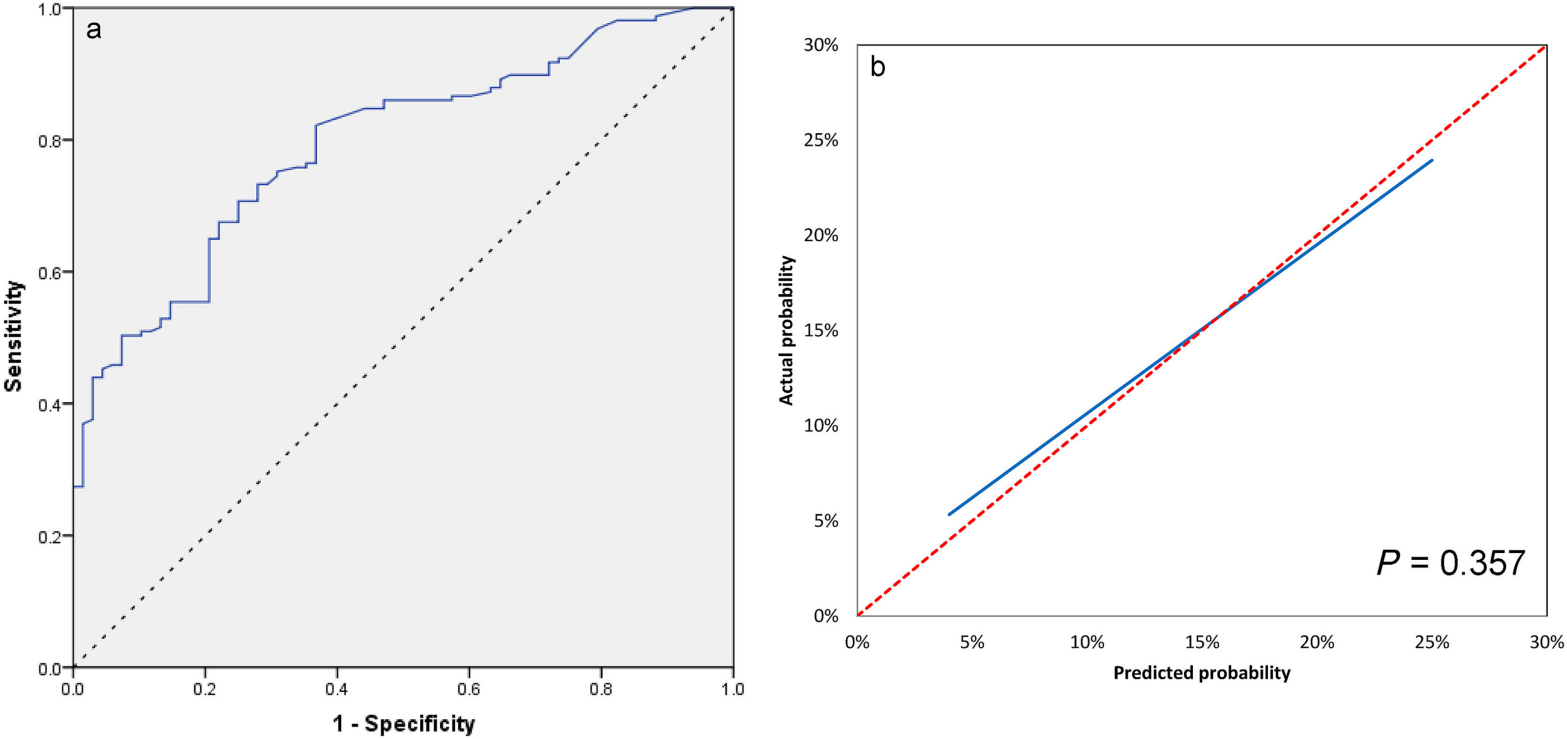
The model ROC (a) and calibration curves (b) of internal validation.

Model ROC curves are shown in Figure 2a. The model had a sensitivity of 70.7% and specificity of 75.0% with the Youden index at 0.457 and area under the curve (AUC) of 0.793 (95% CI: 0.734–0.852). No significant difference was found between predictive and actual numbers (*p* = 0.357, Table 7). Calibration curves indicated good internal stability (Figure 2b).

**TABLE 7:** Verification of the prediction model.

Prediction model	Actual condition		*p*
	Noninvasive	Invasive	
Noninvasive	33	25	0.357
Invasive	35	132	

#### 3.1.6 The Nomogram and the GGN Screening Scale

**FIGURE 3:**
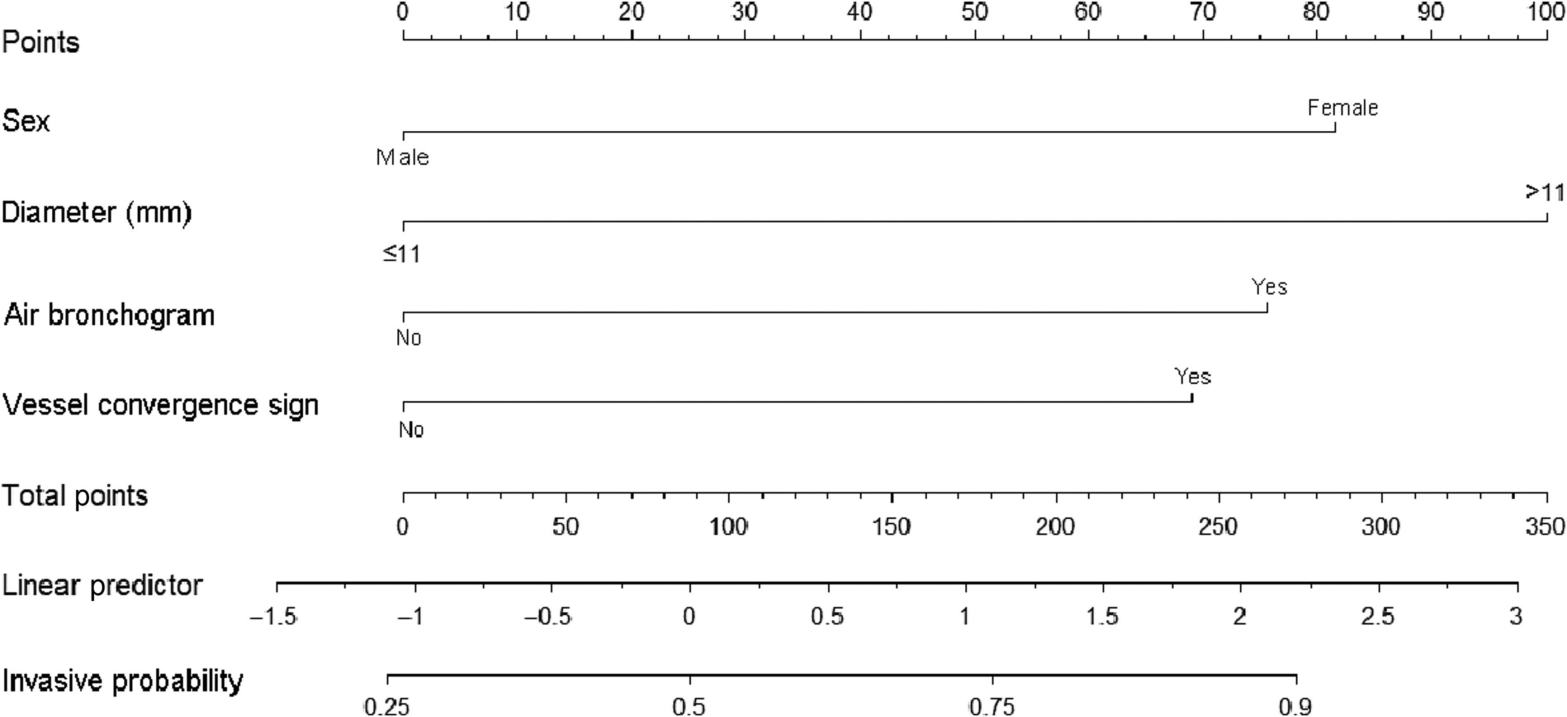
The nomogram of the model to diagnose GGNs.

**FIGURE 4:**
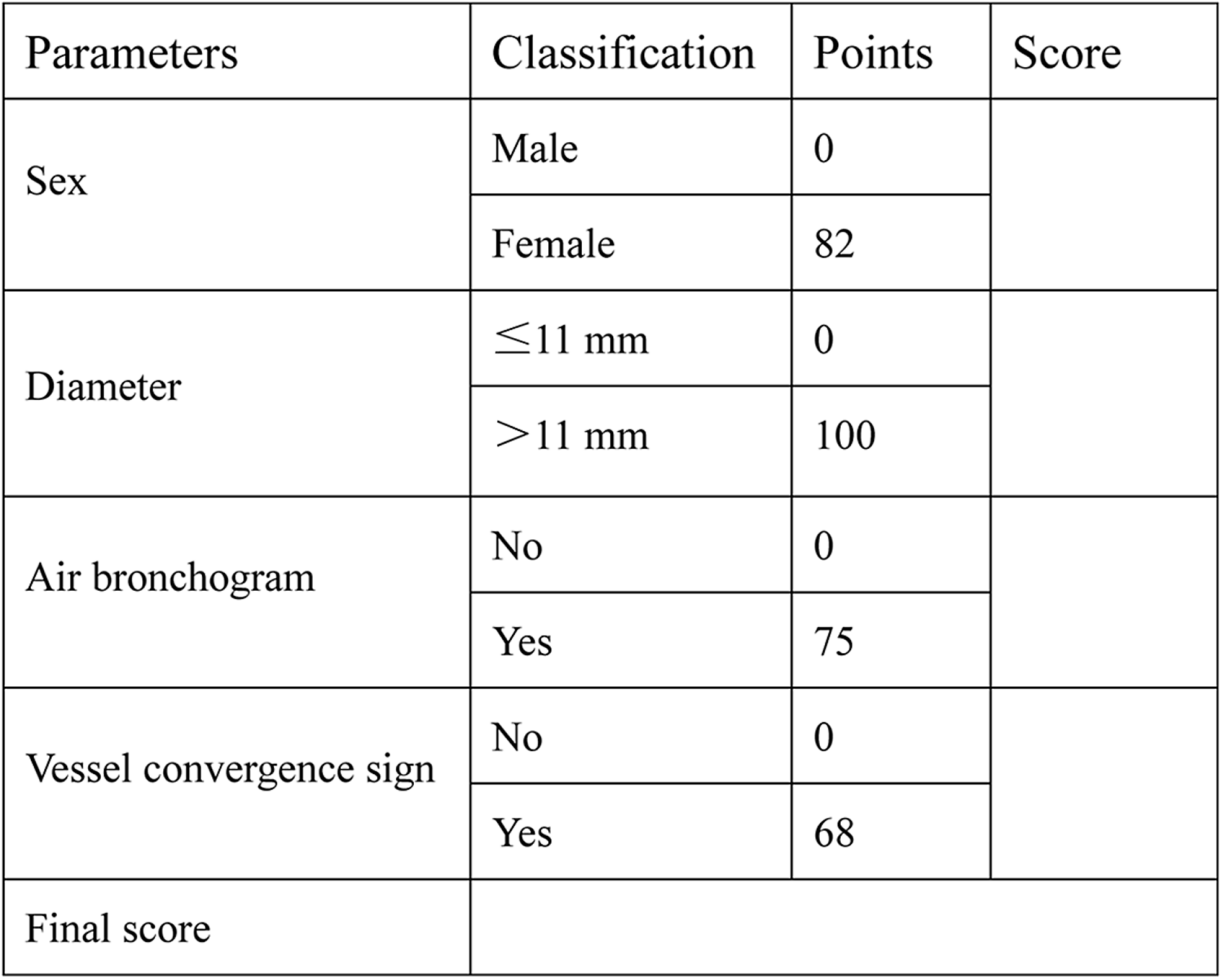
The GGN screening scale.

The GGN screening scale was constructed according to the nomogram and data shown in Figures 3 and 4. The cut‐off point of the final score was 82, based on the first quartile. Therefore, a final score of >82 indicates an invasive GGN and ≤82 a noninvasive GGN.

### 3.2 The Diagnostic Efficiency of the Scale

#### 3.2.1 Sample Size

The sensitivity and specificity for distinguishing invasive GGNs were both 0.773 when the mean CT value > −368.25 HU [22], and the sample size was 68 in the CT‐value group. The sensitivity of the scale group was the same as for the nomogram, giving a sample size of 80 for the scale group.

#### 3.2.2 Patient Characteristics

A total of 147 patients, 59 men and 88 women, with 148 GGNs, ranging in age from 18 to 86 years were recruited between January 2023 and March 2023. No significant differences in sex, age, histopathology, pathological type, location of tumor, or stage were found between the two groups (Table 8).

**TABLE 8:** Comparison of clinical characteristics of patients in the CT‐value and scale groups.

Items	CT‐value (*n* = 68)	Scale (*n* = 80)	*p*
Sex			0.553
Male	27	32	
Female	40	48	
Age (years old)	67.00 ± 11.93	80.00 ± 14.87	0.135
Histopathology			0.305
Benign	4	2	
Adenocarcinoma	61	72	
Squamous	1	5	
Others	2	1	
Pathological type			>0.999
Noninvasive	7	8	
Invasive	61	72	
Location of tumor			0.742
RUL	22	31	
RML	4	4	
RLL	17	16	
LUL	12	18	
LLL	13	11	
Stage			0.625
AAH	3	5	
AIS	0	1	
IA1	23	22	
IA2	23	31	
IA3	6	7	
IB	4	9	
IIB	1	0	
IIIA	4	2	
IV	0	1	

Abbreviations: AAH, Atypical adenomatous hyperplasia; AIS, adenocarcinoma in situ; LLL, left lower lung; LUL, left upper lung; RLL, right lower lung; RML, right middle lung; RUL, right upper lung.

#### 3.2.3 External Validation of the Prediction Model

**FIGURE 5:**
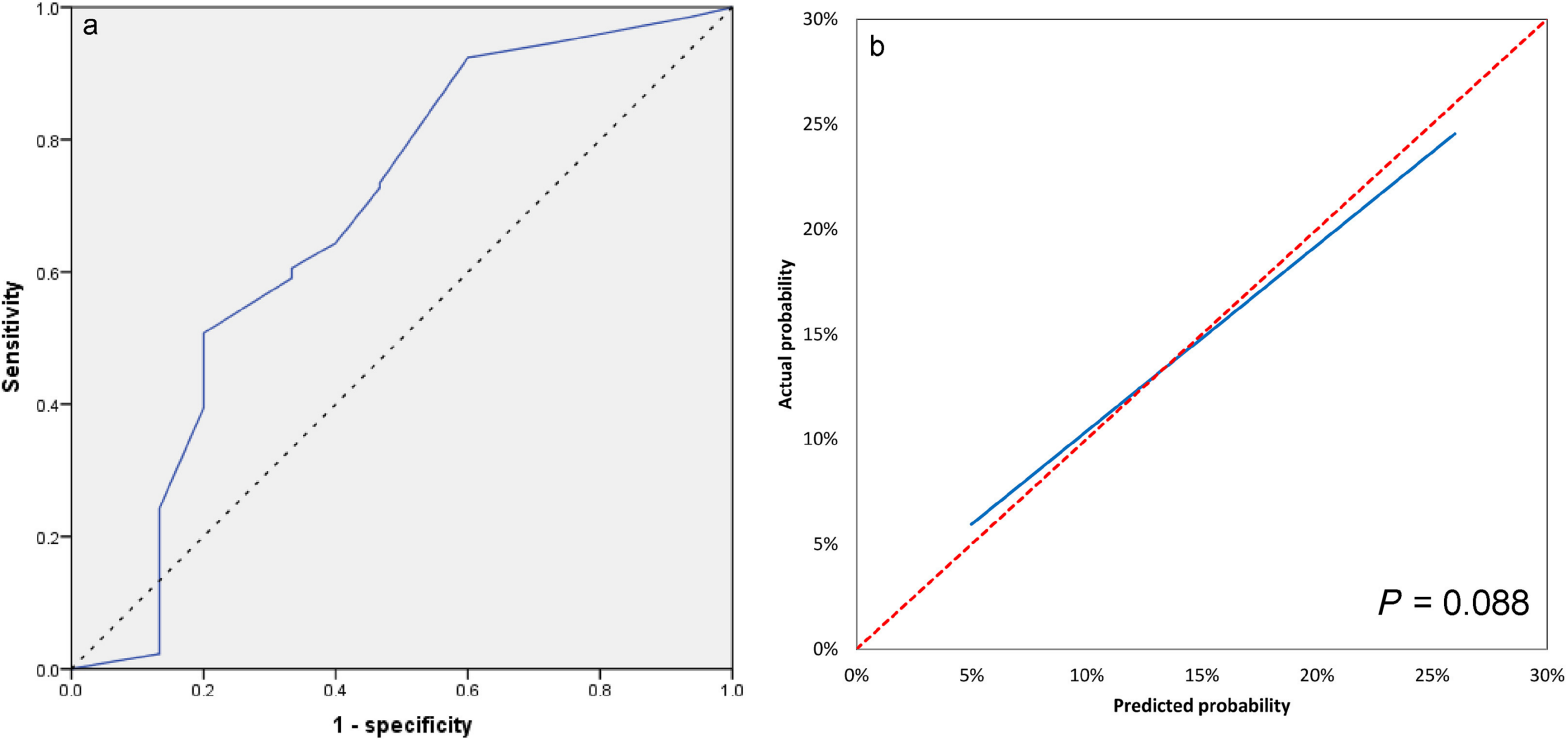
The model ROC (a) and calibration curves (b) of external validation.

External validations of model ROC curves are shown in Figure 5a. The model had a sensitivity of 92.4% and specificity of 40.0% with the Youden index at 0.324 and AUC of 0.678 (95% CI: 0.509–0.847). Calibration curves indicated good external stability (Figure 5b).

#### 3.2.4 Diagnostic Efficiency of the Scale

The false positive rate (FPR) was 62.50%, the false negative rate (FNR) was 25.00% for the scale group, the sensitivity was 75.00%, and the specificity was 37.50%. The positive predictive value (PPV) was 91.53%, and the negative predictive value (NPV) was 14.29% for the scale group. Accuracy was 71.25%. The FPR was 71.43% and the FNR 47.54% for the CT‐value group giving a sensitivity of 52.46% and a specificity of 28.57%. The PPV was 86.49%, and the NPV was 6.45% for the CT‐value group. The accuracy was 50.00%. The confusion matrix for the two groups is shown in Table 9.

**TABLE 9:** The confusion matrix of the two groups.

Pathological biopsy	CT value		Total	Scale		Total
	Noninvasive	Invasive		Noninvasive	Invasive	
Noninvasive	2	5	7	3	5	8
Invasive	29	32	61	18	54	72
Total	31	37	68	21	59	80

## 4 Discussion

It is acknowledged that assessment of GGNs should take into account a patient's age and history, including smoking history, accompanying disease, prior malignancy, and environmental exposure in addition to radiological findings [23]. In the retrospective cohort, the construction of the current scale thus accommodated 20 variables to synthesize clinical, TM, and CT characteristics to evaluate the GGNs, and independent factors were identified.

Age, history of smoking, history of lung disease, family history of lung cancer, and occupational or environmental exposure were found not to be independent predictors of invasive GGNs, whereas sex was confirmed as an independent factor. The cause of most GGNs is adenocarcinoma, which shows a lower positive, linear relationship with smoke inhalation than squamous cell cancer (SCC) or small cell lung cancer (SCLC). Smoking is less frequent among women, causing adenocarcinoma to be a significantly more common lung cancer type in women than men [24, 25]. The five most widely used TMs were not significantly associated with invasive GGNs, perhaps due to low sensitivity in the diagnosis of small tumors [26]. In addition, most GGNs represent adenocarcinoma, whereas NSE and proGRP are specific predictors of SCLC [27] and CYFRA 21‐1 and SCCA of SCC [26, 28]. CEA can be used to monitor adenocarcinoma but is not specific to this tumor‐type since the increase in CEA may also occur in both SCLC and NSCLC [26].

All CT features were found to be significantly different between the two groups of the present study, but only diameter, air bronchogram, and vessel convergence sign were independent predictors of invasive GGNs. A larger lesion diameter is known to increase the likelihood of pathological invasiveness [29]. Jin et al. [30] have identified a cut‐off value of 10.5 mm and Lee et al. [31] 10 mm for the maximum diameter for discrimination between preinvasive and invasive lesions. The current study found a cut‐off value of 11.15 mm. Invasive GGNs were found to be more likely to have an air bronchogram and vessel convergence sign, consistent with the results of Ding et al. [29] and Zhang et al. [6]. Lobulation and spicule signs were not found to be independent factors for invasive GGNs, perhaps due to uncertainty regarding the relationship between pathological invasiveness and lesion shape [29]. Some studies have concluded that pleural indentation is indicative of an invasive lesion [32, 33], but it was not found to be an independent factor for invasive GGNs in the current work. Pleural indentation is usually detected in lesions near pleura and is not obvious in small and low CT attenuation lesions.

Independent predictors of invasive GGNs were amalgamated into a model that was shown to have good discrimination and calibration in both internal and external validation (Figure 3). Previous models have been based on clinical characteristics and ^18^F‐fluorodeoxyglucose positron emission tomography‐CT to identify benign and malignant GGNs, giving an AUC of 0.875, sensitivity of 0.702, and specificity of 0.923 [34]. An alternative used artificial intelligence (AI) and CT signs to identify pathological subtypes of lung adenocarcinoma appearing as GGNs, giving an AUC of 0.779 for identification of AAH/AIS and MIA and 0.918 for MIA and IA [35]. However, the current model is the first to accommodate benign, preinvasive, and invasive lesions. The division of lesions into noninvasive and invasive groups allows surgery to be considered for the latter and surveillance and observation for the former, including cases of AAH or AIS.

The scale was based on the nomogram, and final scores were calculated by adding individual scores of each parameter. Superior results were achieved for PPV (91.53% vs. 86.49%) and sensitivity (75.00% vs. 52.46) by comparison with CT values. A computer‐aided diagnosis (CAD) with a PPV of 54.4% [36] and a deep learning model with a sensitivity of 54.4% [37] have been reported, both of which are lower values than the current scale. The scale achieved an equivalent level of accuracy to a CAD and higher than previously reported visual detection by senior radiologists [38]. Performance on specificity and NPV was poor, although better than CT values (37.50% vs. 28.57% and 14.29% vs. 6.45%, respectively).

Data required for use of the scale are readily available in the clinic, making this mode of assessment accessible to clinicians who are not specialists in thoracic surgery, respiratory medicine, or radiology and allowing its use in primary and less advanced hospitals. The GGN screening scale was based on a prediction model that incorporated comprehensive patient information but also has some refinements. Risk prediction models indicate a probability of a lung nodule being invasive, whereas the screening scale presents a clear cut‐off point above which surgery should be considered. Some prediction models apply to both solid nodules and GGNs, whereas the scale is only appropriate for GGNs. GGNs and solid lung cancers are fundamentally different diseases [2], and adding solid lesions to the scale would compromise its utility for GGN assessment. Furthermore, patients who have lung nodules but for whom surgery is not an option may be eligible for radiofrequency ablation (RFA) and assessment via the current scale may inform such treatment decisions. Lesions are normally confirmed as AAH, AIS, or MIA by biopsy prior to RFA [39], but the scale may prove to be an alternative. In addition, the scale indicates the need for surgical intervention rather than merely indicating a benign or malignant nature. Some cases of AAH and AIS do not require immediate surgery despite their malignant nature.

We acknowledge some limitations to the current study. First, the screening scale was based on a prediction model. A different cut‐off point may have been identified for a different sample size or clinical characteristics. Second, the criterion for positive nodules in the CT‐value group was a mean CT value > −368.25 HU, and changing this cut‐off point may have led to nonsignificant results. Third, the number of parameters used to construct the scale was small and would be improved by expanding the sample size in the first stage. Fourth, the specificity and NPV were better than those for CT assessment but were less than satisfactory, perhaps due to the low number of parameters. Improvements would be achieved by modifying the scale or serial testing to increase specificity.

In summary, this is the first report of a scale for screening of GGNs and represents a diagnostic aid for lung cancer. Its high sensitivity and accuracy make it suitable for primary screening. Invasive GGNs may thus be treated in a timely and specialized manner. Future studies on follow‐up of GGNs are planned, as is a screening scale for solid lung nodules and improvements using AI algorithms.

## 5 Conclusion

The GGN screening scale has high sensitivity and accuracy with low specificity and NPV and is suitable for primary screening. A final score of ≤82 may be an indication for surveillance and observation, and surgery should be carried out if the final score is > 82.

## Author Contributions

Lei bi and Minhao Yu concepted and designed the study. Lei bi defined intellectual content. Minhao Yu and Yalin Cheng prepared and wrote the manuscript. Tao Wen, Liming Zhang, Xiubo Wei, Yonghong Wang, Jiang Du, and GuangKe Xie collected and analyzed the data and proceed the statistical analysis. All authors reviewed the manuscript.

## Conflicts of Interest

The authors declare no conflicts of interest.

## Data Availability

The datasets generated and analyzed during the current study are available in the *Research Manager* repository (http://www.medresman.org.cn/pub/cn/proj/searchSH.aspx).
